# Supplementary material for: Complexome profiling of the Chlamydomonas psb28 mutant reveals TEF5 as an early PSII assembly factor
Source: Plant Cell. 2025 Mar 17;37(6):koaf055. doi: 10.1093/plcell/koaf055 (PMC12164590; doi:10.1093/plcell/koaf055)
Supplement: koaf055_Supplementary_Data [file koaf055_supplementary_data.zip › psb28_interactive.zip!/plots/intensity/qualityPlots/peptides/PETO_Cre12.g558900.t1.2.html]

### Cre12.g558900.t1.2

Trivial name: PETO  
SeqCoverage [median]: 42.90  
Mass: 20.25 kDa  
  
 

| Sequence | MissedCleavage | Mass [Da] | wtIntensity | muIntensity | Score |
| --- | --- | --- | --- | --- | --- |
| AEVVESTSGGLDPR | 0 | 1415.69 | 448.80 | 883.35 | 119.45 |
| AFQDDTPAAPPPK | 0 | 1353.66 | 2139.11 | 3174.84 | 188.28 |
| AFQDDTPAAPPPKR | 1 | 1509.76 | 90034.75 | 61608.48 | 180.48 |
| AFQDDTPAAPPPKREFR | 2 | 1941.97 | 8322.62 | 7689.08 | 136.34 |
| DSNNYAGYEATLK | 0 | 1444.65 | 69438.11 | 58583.64 | 241.39 |
| DSNNYAGYEATLKSENGVTFPK | 1 | 2404.12 | 1563.31 | 0.00 | 54.15 |
| GLAPPALPQSDAPNVAAEKPK | 1 | 2070.11 | 219137.47 | 239740.16 | 147.10 |
| KAEVVESTSGGLDPR | 1 | 1543.78 | 266056.72 | 160098.57 | 144.72 |
| KAFQDDTPAAPPPK | 1 | 1481.75 | 320.74 | 477.97 | 60.63 |
| KAFQDDTPAAPPPKR | 2 | 1637.85 | 21286.41 | 7160.07 | 99.94 |
| KAFQDDTPAAPPPKREFR | 3 | 2070.06 | 1552.03 | 0.00 | 86.77 |
| KGGFPFGGK | 1 | 893.48 | 881.34 | 4469.07 | 106.36 |
| SENGVTFPK | 0 | 977.48 | 15075.43 | 21317.68 | 162.88 |
|  |  |  | 696256.8 | 565202.9 |  |
